# Design and validation of a deep learning GAN for predicting ^1^ ^7^ ^7^Lu dose voxel kernels using GATE/GEANT4 Monte Carlo and IDAC‐Dose 2.1 in phantom dosimetry

**DOI:** 10.1002/acm2.70661

**Published:** 2026-06-15

**Authors:** Otieno Erick Kapis, Muchai Ian Kaniu, Iaccarino Giuseppe, Hudson Kalambuka Angeyo

**Affiliations:** ^1^ Department of Physics University of Nairobi Nairobi Kenya; ^2^ Department of Radiation Oncology Cancer Treatment Center The Nairobi Hospital Nairobi Kenya; ^3^ Laboratory of Medical Physics IRCCS Istituto Nazionale Tumori Regina Elena Rome Italy

**Keywords:** 3D‐GAN DVK Model, GATE Monte Carlo, Lu‐177 DVK Dosimetry

## Abstract

**Background:**

The rapid evolution of deep learning (DL), particularly the emergence of generative adversarial networks (GANs) has demonstrated strong potential for generating high‐resolution, voxel‐level dosimetric data. However, GAN‐based approaches have not yet been broadly applied to the generation of tissue‐specific dose voxel kernels (DVKs) in internal dosimetry. This gap underscores a critical unmet need for methods that can deliver Monte Carlo–level accuracy while achieving substantially improved computational efficiency.

**Purpose:**

This study aimed to design a GAN architecture to train models that can generate ^177^Lu DVKs for internal radiation dosimetry comparable to Monte Carlo standards.

**Materials and methods:**

A GAN model was trained with paired CT‐derived density kernel maps (DKs; voxel size 0.24^3^ cm^3^, matrix 15 × 15 × 15) and their corresponding Monte Carlo‐simulated ^177^Lu DVKs. The GAN‐generated DVKs were applied to SPECT images of spherical phantoms with varying volumes and initial ^177^Lu activity concentrations to compute mean absorbed doses. Results were validated against full GATE Monte Carlo simulations and IDAC‐Dose 2.1 application.

**Results:**

The GAN model predicted ^177^Lu DVKs for water, soft tissue, muscle, and cortical bone with accuracies of 98.2%, 98.3%, 96.9%, and 94.2%, respectively (*p* > 0.05). Comparisons of GAN‐derived ^177^Lu DVKs with both GATE Monte Carlo and IDAC‐Dose 2.1 produced very strong correlations (Pearson's *r* > 0.99; *p* = 0.0001). Bland–Altman analysis showed narrow limits of agreement at the 95% confidence interval (CI: −0.59 Gy to 0.44 Gy for GATE MC vs. GAN DVK, and CI: −0.01 Gy to −0.21 Gy for IDAC‐Dose 2.1 vs. GAN DVK), with the vast majority of points falling within these ranges. These findings confirm the robustness and reliability of the GAN‐predicted DVKs when benchmarked against established dosimetric methods.

**Conclusion:**

GAN‐generated ^177^Lu DVK convolution provides a potentially fast and computationally efficient approach for internal dosimetry, with strong agreement to established methods in phantom studies. While the clinical impact of tissue‐specific DVKs remains dependent on broader improvements in quantitative imaging and dose–response characterization, this approach represents a promising proof‐of‐concept toward more physically consistent and scalable voxel‐level dosimetry.

## 1 INTRODUCTION

Accurate internal dosimetry is fundamental to personalized targeted molecular therapy, where precise estimation of absorbed dose determines both treatment efficacy and safety.^1^ Traditional dosimetry methods such as the Medical Internal Radiation Dose (MIRD) committee formalism and the IDAC‐Dose 2.1 application calculate absorbed doses using organ‐level S‐values derived from standardized reference phantoms.^2^, ^3^ Although these approaches are computationally efficient, they assume uniform activity and energy deposition within organs, leading to potential inaccuracies in heterogeneous or patient‐specific geometries.^3^ In contrast, voxel‐level dosimetry using dose voxel kernels (DVKs) or dose point kernels (DPKs) enables high‐resolution, spatially resolved dose estimation by convolving activity maps with precomputed energy‐deposition kernels.^4^, ^5^ However, accurate computation of DVKs and DPKs using Monte Carlo (MC) transport codes remains computationally demanding, limiting their clinical translations.

Among therapeutic radionuclides, Lutetium‐177 (^1^ ^7^ ^7^Lu) has emerged as one of the most clinically important isotopes in theranostic applications, combining both diagnostic imaging and targeted radionuclide therapy.^6^, ^7^ Its favorable physical properties include; medium‐energy β^−^ emissions (∼0.5 MeV) suitable for localized tumor irradiation, and γ emissions at 113 and 208 keV that enable imaging, make it particularly effective for treating neuroendocrine tumors and prostate cancer through agents such as ^1^ ^7^ ^7^Lu‐DOTATATE and ^1^ ^7^ ^7^Lu‐PSMA‐617 respectively.^8^, ^9^ Owing to its growing clinical importance, the development of computationally efficient and patient‐specific dosimetry tools for ^1^ ^7^ ^7^Lu‐based or other targeted radionuclide therapies is increasingly important for preclinical and methodological studies aimed at future patient‐specific applications.

Recent advances in deep learning (DL) and artificial intelligence (AI) have shown promise in accelerating MC‐based dose prediction by learning the complex mapping between anatomical images and dose domains.^10^, ^11^ Convolutional neural networks (CNNs) and deep neural networks (DNNs) have been widely employed to predict three‐dimensional (3D) dose maps or energy deposition patterns from imaging and treatment‐planning data.^11^, ^12^ For example, Mentzel et al. proposed a conditional 3D U‐Net generative adversarial network (GAN) that accurately predicted voxel‐wise energy deposition in heterogeneous phantoms, while Kearney *et al*. introduced DoseGAN, an attention‐gated GAN that generated realistic radiotherapy dose distributions.^11^, ^12^ DoseGAN demonstrated superior performance in predicting realistic volumetric dosimetry compared to all other evaluated algorithms, with statistically significant improvements observed across all comparative analyses. Similarly, Scarinci et al. and Akhavanallaf et al. applied machine learning models to calculate dose point kernels and voxel S‐values respectively, illustrating the capability of AI to emulate MC‐based dosimetry.^13^, ^14^

Studies shows that GANs provide a key advantage over conventional CNNs trained with voxel‐wise regression losses like mean square error (L1), which primarily optimize numerical agreement but may fail to preserve fine spatial structure, as seen in prior DVK prediction work using supervised CNN frameworks.^15^ In contrast, GANs incorporate an adversarial loss through a discriminator that evaluates the realism of generated outputs, encouraging the model to learn the underlying data distribution and produce spatially coherent, physically consistent patterns. This approach has been shown to improve the reconstruction of high‐frequency features and reduce over‐smoothing commonly observed in regression‐based CNNs, particularly in tasks involving complex spatial fields such as dose distributions.^11^, ^16^, ^17^

Despite these advances in GAN, no published work has yet shown the use of GANs in predicting ^1^ ^7^ ^7^Lu DVKs directly. Most of the GAN‐based studies focus primarily on global dose distributions, without addressing the kernel‐based dose transfer functions that underpin voxelized dosimetry. This represents a critical knowledge gap, as DVKs form the foundation for rapid convolution‐based absorbed dose calculations across tissues with varying densities and compositions.

In this study, we developed and validated a 3D DL GAN model trained on reference ^1^ ^7^ ^7^Lu DVKs generated from CT‐derived density kernels (DKs) using the EGSnrc/DOSXYZnrc Monte Carlo code. EGSnrc is a gold‐standard simulation toolkit extensively validated for radiation transport, electron–photon interactions, and internal dosimetry, providing highly accurate sub‐millimeter energy deposition estimates that serve as reliable ground truth for data‐driven modeling.^18^ The proposed GAN model is trained on a large and diverse set of CT‐derived DKs to learn a generalized mapping between heterogeneous density patterns and their corresponding radiation transport in DVKs. This strategy enables patch‐wise DVK prediction for spatially varying and previously unseen tissue configurations, rather than assigning a single representative DVK per tissue class. Such generalization is essential for capturing local density variations and tissue interfaces present in real anatomical structures, while also reducing the risk of overfitting. The GAN‐predicted ^1^ ^7^ ^7^Lu DVKs are subsequently convolved with time‐integrated activity (TIA) maps derived from ^1^ ^7^ ^7^Lu SPECT images of spherical phantoms to generate absorbed dose distributions. However, it should be noted that extending this approach to a different radionuclide would still require retraining the GAN model, similar to direct activity‐to‐dose prediction frameworks. The key distinction, however, is that the GAN‐DVK approach explicitly decouples the radiation transport physics from the activity distribution.

The validity of the GAN‐predicted DVKs is evaluated through cross‐comparison with (i) voxel‐level Monte Carlo dose maps from GATE/GEANT4 simulations and (ii) organ‐level mean absorbed dose estimates from IDAC‐Dose 2.1 based on the MIRD formalism. This framework bridges voxel‐wise and organ‐level dosimetry and introduces a physics‐informed, computationally efficient approach for scalable DVK generation, supporting the development of more consistent and individualized internal dosimetry workflows.

## 2 MATERIALS AND METHODS

### 2.1 DVKs simulation and GAN model training

**FIGURE 1:**
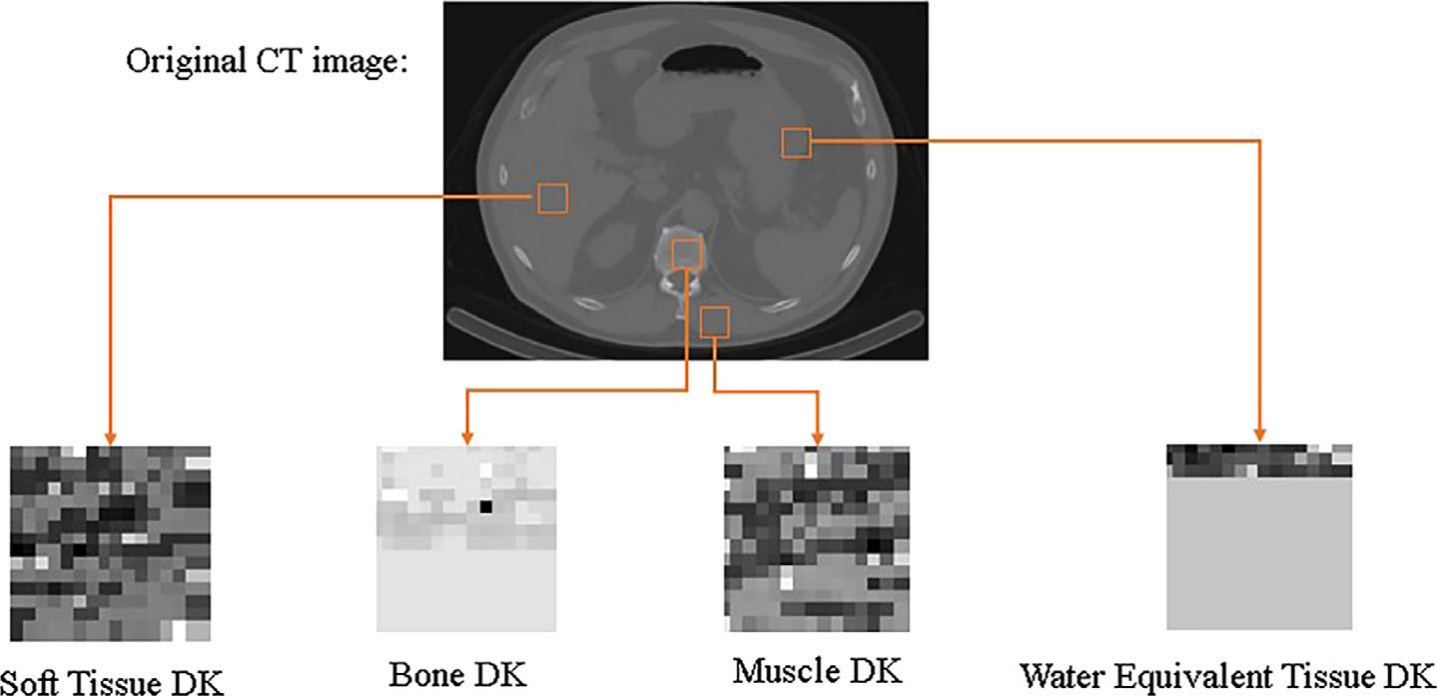
Density Kernel maps cropped from selected tissues of patient CT image.

Monte Carlo simulations were conducted using the EGSnrc/DOSXYZnrc radiation transport code to generate reference ^177^Lu DVKs for training the DL GAN models. The simulation geometry was constructed from anonymized patient CT images retrieved from a public repository.^19^ The CT images were cropped into small cubic sub‐volumes, termed as DKs as presented in Figure 1. Each DK consisted of 15 × 15 × 15 voxels with an isotropic voxel size of 0.24^3^ cm^3^, representing a physical region of 3.6 cm per side. This kernel dimension is sufficient to fully capture the short‐range β^−^ energy deposition of ^1^ ^7^ ^7^Lu (maximum range ∼1.5–2 mm), which dominates the absorbed dose.^7^ Although the longer‐range γ emissions (113 and 208 keV) may extend beyond the kernel boundaries, their contribution to the total dose is minimal, as evidenced by the fact that the central voxel accounts for over 90% of the deposited energy within the kernel. Although the volume‐averaged DVK uncertainty was low, 0.93%, evaluation of the fractional contribution of the gamma emissions would however be necessary for completeness of the DVK kernel characterization.

These DK sub‐volumes were extracted from CT regions corresponding to key organs of interest, including kidney, liver, bone, muscle, and soft tissue. The voxel intensities (in Hounsfield Units) were converted to material densities using a CT‐to‐density calibration curve based on the empirical calibration approach described by Schneider *et al.* to ensure physically accurate mass‐energy transport parameters within DOSXYZnrc.^20^ The diversity of the training dataset of heterogeneous DKs, is critical to ensure model generalization and to prevent overfitting, particularly given the variability of tissue composition in CT images.

The central voxel of each DK was defined as the source region, containing an isotropic ^1^ ^7^ ^7^Lu emission modeled as a parallelepiped source. The radionuclide's β^−^ and γ emissions were simulated using transport thresholds of ECUT = 0.521 MeV and PCUT = 0.10 MeV. These cutoffs provide a practical balance between computational efficiency and physical accuracy for simulating electron transport and photon interactions at the sub‐centimeter scale, consistent with established Monte Carlo dosimetry practices.^21^ A total of 5.0 × 10^8^ primary particle histories were simulated per kernel, yielding voxel‐level statistical uncertainties below 2%, which is consistent with standard Monte Carlo dosimetry precision limits.^4^ The resulting ^1^ ^7^ ^7^Lu DVK dose distributions were expressed in Gray per disintegration. The Monte Carlo‐derived ^1^ ^7^ ^7^Lu DVKs kernels served as ground‐truth datasets for supervised training of 3D‐GAN model. Both the DKs and DVKs were preprocessed by edge padding to matrix size 16 × 16 × 16 for input in GAN network.

### 2.2 3D GAN design

**FIGURE 2:**
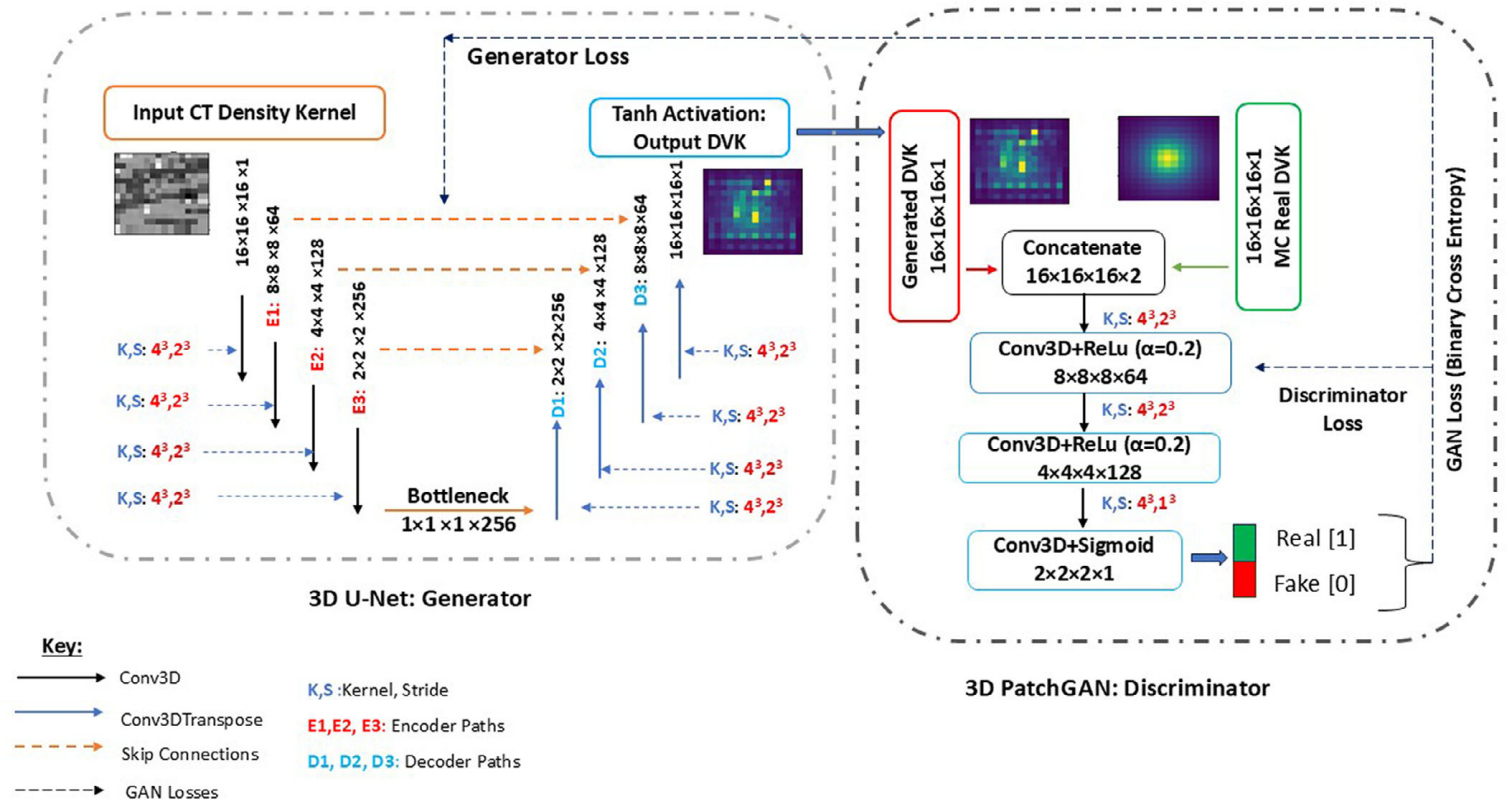
3D GAN architecture showing the generator and 3D PatchGAN discriminator.

The new 3D‐GAN architecture presented in Figure 2 was custom developed in python and adapted from the 2D Pix2Pix framework.^17^ It consists of a 3D U‐Net generator and a 3D PatchGAN discriminator.^12^, ^17^ The generator performs voxel‐to‐voxel translation between DKs and their corresponding ^1^ ^7^ ^7^Lu DVKs, while the discriminator evaluates the realism of generated DVK outputs. The generator follows an encoder‐decoder topology with three down‐sampling convolutional layers (filters: 64, 128, 256) followed by a bottleneck layer and three symmetric up‐sampling transposed convolutions (filters: 256, 128, 64). *LeakyReLU* and *ReLU* activations were used for encoding and decoding layers, respectively, with batch normalization and dropout (rate = 0.5) applied to stabilize learning. Skip connections were applied to preserve fine spatial detail, and a *tanh* activation in the final layer ensures outputs normalized between −1 and 1.

The discriminator employs a 3D PatchGAN architecture, in which real/fake decisions are made over local cubic regions. Based on the sequence of convolutional kernel sizes, strides, and network depth, the theoretical receptive field is 22 × 22 × 22, while the physical input DVK patch size is 16 × 16 × 16. Because all Conv3D layers use “same” padding, TensorFlow/Keras implicitly applies zero‐padding at the input boundaries during convolution. Consequently, the portion of the receptive field extending beyond the 16 × 16 × 16 DVK patch does not correspond to additional DVK information but instead covers zero‐valued padded regions introduced internally by the convolution operation.

The network processes concatenated input–target pairs (16 × 16 × 16 × 2) through successive 3D convolutional layers (64, 128, and 1 filters) with *LeakyReLU* activations and batch normalization, producing a voxel‐wise probability map via sigmoid activation.

### 2.3 Adversarial training

The generator (G) and discriminator (D) were jointly optimized within a conditional adversarial framework, where the generator aimed to synthesize DVKs that were indistinguishable from MC‐simulated ground truths. Conversely, the discriminator was trained to distinguish between real and generated DVKs. During generator updates through generator loss feedback, the discriminator's weights were frozen to enhance stability and prevent feedback oscillations. The total objective combined an adversarial loss term that encouraged realistic DVK generation with an L1 reconstruction loss that enforced voxel‐wise agreement with MC data. The training objective was expressed in Equations (1), (2), (3).^17^

(1) $$L_{\mathrm{total}}=L_{\mathrm{adv}}+\lambda L_{L1}$$

where

(2) $$L_{\mathrm{adv}}=E_{x,y}\left[\log D\left(x,y\right)\right]+E_{x}\left[\log\left(1-D\left(x,G\left(x\right)\right)\right)\right]$$

represents the binary cross‐entropy adversarial component, and

(3) $$L_{L1}=E_{x,y}\left[||y-G\left(x\right)|{|}_{1}\right]$$

quantifies the voxel‐wise mean absolute error (MAE) between generated and reference DVKs.

**FIGURE 3:**
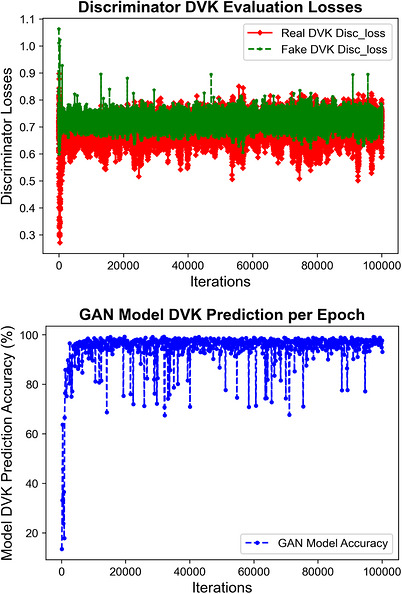
In (a) is discriminator binary classification losses on real and fake DVKs while (b) is the GAN model prediction accuracies of 177Lu DVKs at end of each epoch.

The weighting factor λ = 100 was empirically chosen to balance physical accuracy and adversarial realism, emphasizing precise dose reconstruction while preserving structural fidelity. Both networks were trained using the Adam optimizer (β_1_ = 0.5, learning rate = 2 × 10^−^^3^) for 100 000 iterations, each with a batch size of 1 from 3200 dataset. The D binary cross‐entropy losses on accurate classification of both fake and real DVKs are presented in Figure 3(a). The losses were initially higher at the beginning of training but finally settled between 0.6 and 0.8 indicating a balanced adversarial state with no one network dominating. Training was terminated after 49 hours 23 minutes upon reaching Nash equilibrium. The GAN models were generated after every epoch (123 iterations) and their accuracies were evaluated by predicting the ^177^Lu DVKs of randomly picked DKs during training as presented in Figure 3(b).

The high performing trained GAN model was applied to predict ^1^ ^7^ ^7^Lu DVKs for various tissue types. Prediction accuracy was assessed by comparing the voxel values of the predicted DVKs with the corresponding Monte Carlo calculated reference DVK using Equation 4. Accuracy was quantified as the fraction of voxels whose predicted dose values fell within an absolute tolerance of 10^−^^1^ ^5^ mGy/(MBq·s) relative to the reference. Given that voxel dose values span a wide range, from 1 × 10^−^^9^ to 1 × 10^−^^1^ ^7^ mGy/(MBq·s), this tolerance strikes a balance: it accommodates numerical noise in low‐dose voxels while remaining stringent in high‐dose regions.

(4) $$DVK_{predict}\left(\%\right)=\frac{100}{N}\sum_{i=1}^{N}\left[|x_{i}-y_{i}|\leq 10^{-15}\right]$$

Here, $x_{i}$ and $y_{i}$ are the voxel values from the reference and predicted DVKs, respectively, N is the total number of voxels in the kernel, and $10^{-15}$ mGy/(MBq·s) represents the absolute tolerance applied to each voxel comparison.

### 2.4 Activity masking of SPECT and generation of CT images

Spherical digital phantoms were generated as SPECT images and masked with varying activities of ^1^ ^7^ ^7^Lu to mimic real patient SPECT acquisitions at: 0, 24, 72, and 168 hours post injection. The images consist of 164 axial slices with a matrix size of 512 × 512. The activity distributions were created using *MIM Maestro™* (Version 6.8; MIM Software Inc., Cleveland, OH, USA).^22^ Two sets of spherical phantoms were defined: the first comprised 10 spheres of equal volume (500 mL) but with differing initial activities ranging from 600 MBq to 2600 MBq, while the second contained spheres of varying volumes (40 to 3000 mL) all initialized at 2200 MBq. A custom Python program was developed to generate CT DICOM images matching the spatial dimensions of the SPECT images, using GEANT4 material density data^23^ as the basis for tissue representation, as summarized in Table 1. To characterize the temporal activity distribution, the TIA maps were determined by multiplying the initial sphere activity (at time zero) by the corresponding residence time (TIA = 1.44 × $T_{1/2}\times A_{o\;}$, where $A_{o\;}$ is the initial activity and $T_{1/2}$ is the physical half‐life), assuming physical decay only.

**TABLE 1:** GEANT4 based CT tissue densities and GAN 177Lu DVKs prediction accuracies.

Tissue Media	ρ(g/cm^3^)	HU Range	GAN DVK Accuracy (%)	Per voxel *t*‐test (*p*‐value)
G4_water	0.998	−7, 7	98.2	0.24
G4_tissue_soft_icrp	1.03	3, 45	98.3	0.25
G4_muscle_skeletal_icrp	1.05	20, 87	96.9	0.28
G4_bone_cortical_icru	1.85	200, 800	94.2	0.14

### 2.5 Sphere mean dose calculations

The mean absorbed doses to the spherical phantoms were estimated using three complementary approaches: (i) voxel‐wise convolution of the GAN‐ predicted ^177^Lu DVKs, (ii) full Monte Carlo (MC) simulation, and (iii) the organ‐based IDAC‐Dose 2.1 application. The IDAC‐Dose 2.1 framework, based on ICRP Publication 133 reference phantoms, provides standardized internal dosimetry comparable to OLINDA/EXM, and its built‐in sphere model was used for mean absorbed dose calculations.^24^, ^25^

Monte Carlo simulations were performed using a GATE/GEANT4‐based Python implementation.^26^ CT DICOM datasets for four different tissue media and corresponding SPECT spherical phantom images at initial activities were converted into *mhd/raw* formats using the *vv* 4D slicer application.^27^ The *G4EmStandardPhysics_option3* physics list was applied with 1 mm particle production cuts, and the resulting absolute dose distributions served as the reference standard for validation.

GAN‐predicted DVKs for each CT medium were convolved with each of the SPECT images at initial activities, and the results were compared against reference MC and IDAC‐Dose 2.1 calculations. Bland–Altman plots, Pearson correlation coefficients, and paired *t*‐tests were used to quantify agreements and identify systematic deviations. Boxplots were further employed to visualize inter‐tissue variability and data dispersion. Statistical significance was assessed using reported p‐values to evaluate differences between the GAN‐based convolution and MC‐derived absorbed doses.

## 3 RESULTS

**FIGURE 4:**
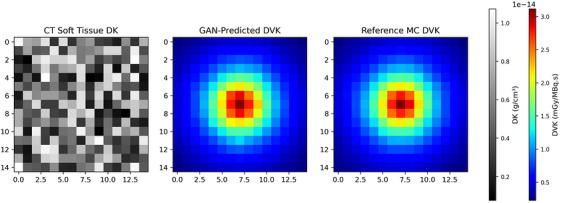
From left; CT soft tissue (15, 15, 15) density kernel, GAN‐predicted DVK and the Monte Carlo calculated reference DVK for the soft tissue.

The trained GAN model was applied to predict ^177^Lu DVKs corresponding to CT‐based DKs representing water, soft tissue, muscle, and bone. Figure 4 illustrates a representative 2D slice of the GAN‐predicted ^177^Lu DVK for a CT soft‐tissue DK. A strong visual correspondence is observed between the GAN‐generated DVK and the reference Monte Carlo‐simulated target ^177^Lu DVK, indicating that the model successfully learned the spatial dose distribution patterns characteristic of the tissue type.

**FIGURE 5:**
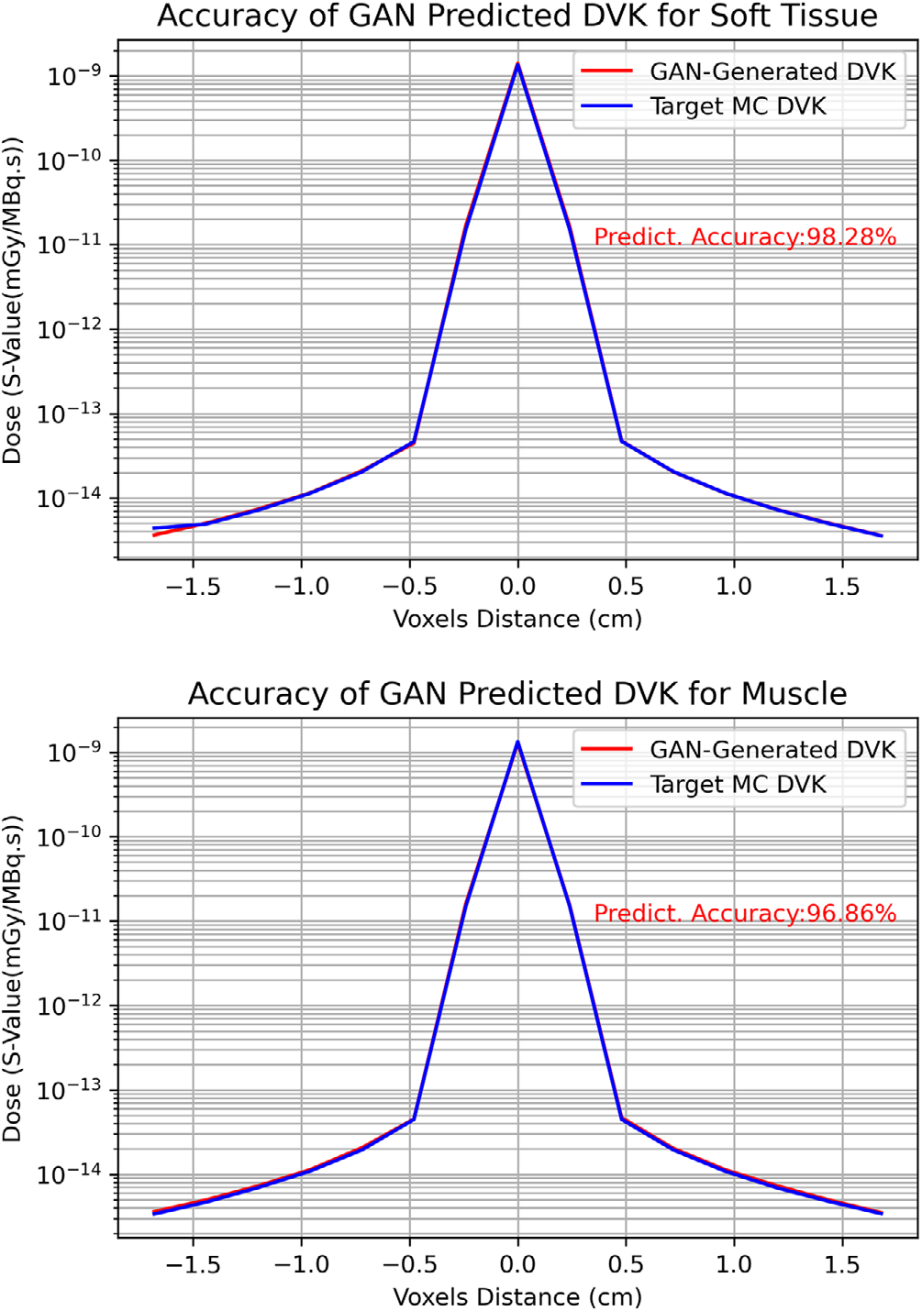
2D profiles, from left; the 177Lu DVK prediction accuracy of GAN model on soft tissue and muscle media respectively.

The comparison between the GAN‐predicted and Monte Carlo‐simulated DVKs demonstrated strong statistical agreement. The *t*‐test yielded p‐values > 0.05, and the 95% confidence intervals of the voxel values mean differences included zero, indicating no statistically significant difference between the two methods. The GAN predictions achieved percentage accuracies exceeding 94% (Table 1), underscoring the model's reliability and consistency relative to the Monte Carlo gold standard. Figure 5a,b present midline profiles of the GAN‐predicted and corresponding reference ^177^Lu DVKs for soft tissue (98.28%) and muscle (96.86%), respectively, both showing a near superposition.

Table 1 summarizes GEANT4 based CT media densities, HU range and the prediction accuracies by the GAN‐model for ^177^Lu DVKs in comparison with the reference Monte Carlo‐calculated ^177^Lu DVKs. Higher accuracies were achieved for water and soft‐tissue DKs, whereas slightly lower values were observed for skeletal muscle and bone. The reduced performance in these latter tissues is attributed to their intrinsic heterogeneity‐bone interfaces with softer components such as bone marrow, while muscle often borders low‐density tissues, introducing localized variations that affect DVK estimation accuracy.

### 3.1 Spherical phantom dose calculations

**FIGURE 6:**
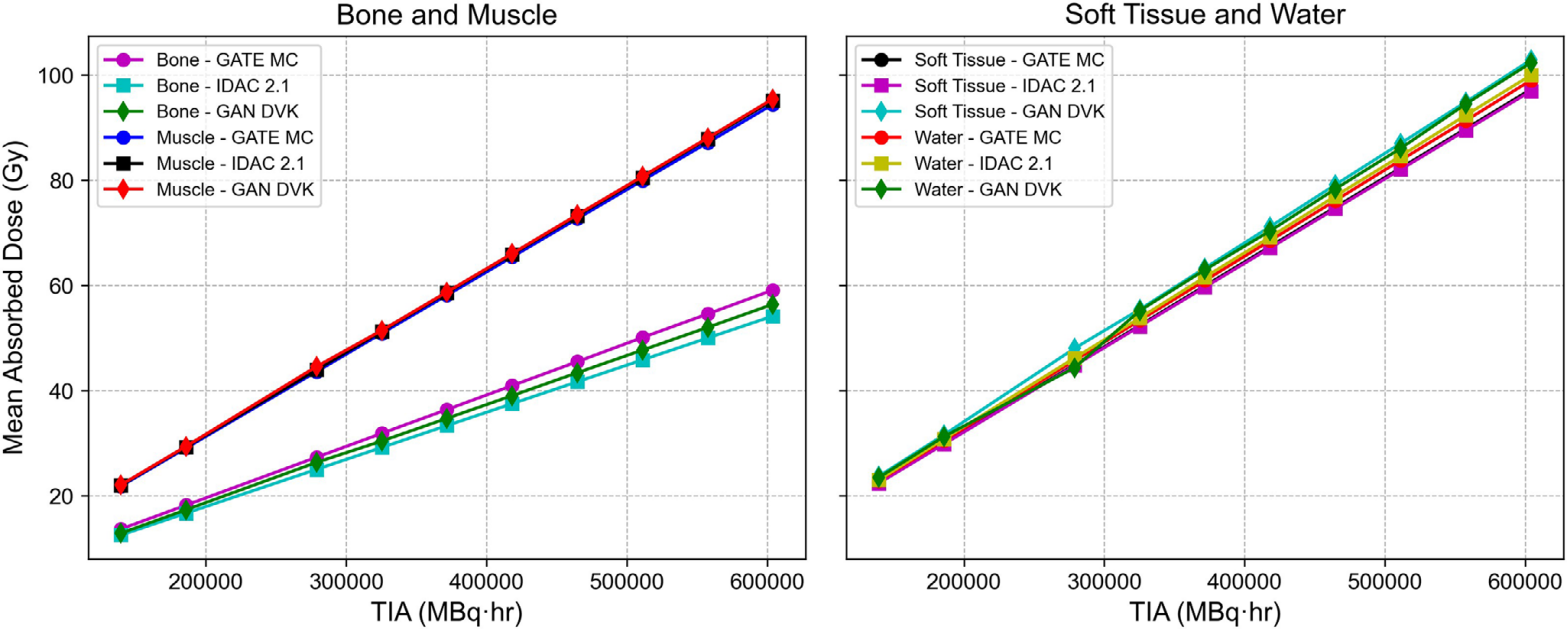
500 mL spherical phantoms absolute doses comparison between GAN‐DVK, GATE MC and IDAC Dose 2.1 calculation methods for different tissues.

The absolute mean doses for 500 mL phantoms derived from the GAN‐predicted ^1^ ^7^ ^7^Lu DVKs for water, soft tissues, bone and muscle exhibited strong statistical agreement with both the GATE Monte Carlo simulations and the IDAC‐Dose 2.1 calculations. Figure 6 illustrates the relationship between the mean absolute absorbed doses in bone and muscle, soft tissues and water respectively, and the corresponding time‐integrated activities (TIA). At lower TIA values, the GAN‐predicted DVKs were indistinguishable from both GATE MC and IDAC‐Dose 2.1. At higher TIA values, the GAN DVKs aligned more closely with GATE MC than with IDAC‐Dose 2.1. These results demonstrate the consistency of the GAN‐based predictions with reference voxel‐based dosimetry and highlight their methodological advancement over traditional organ‐based methods.

**FIGURE 7:**
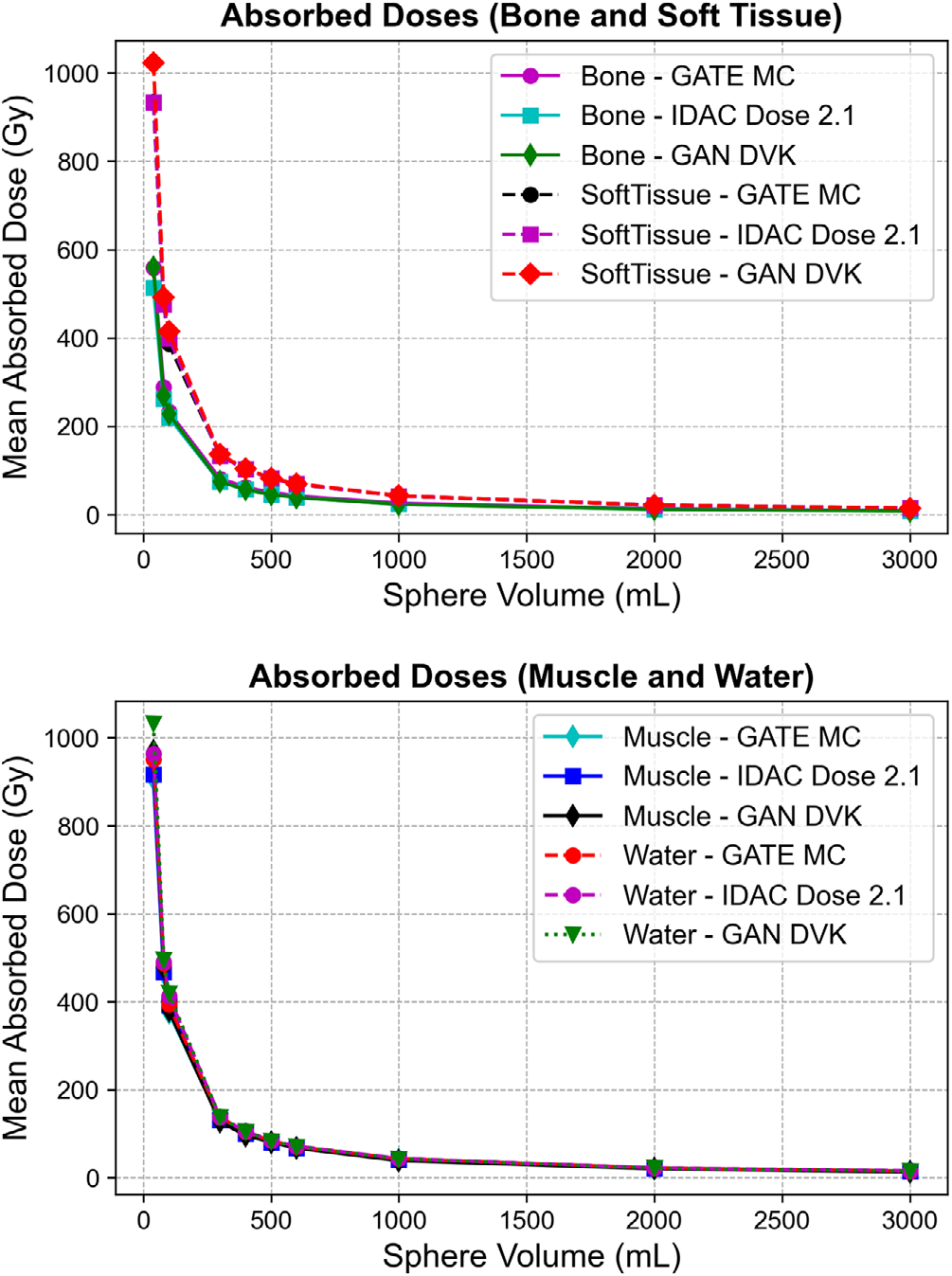
Mean absorbed dose comparison between GATE MC, IDAC, and GAN predicted DVK for (a) bone and soft tissue and (b) muscle and water in phantom studies.

The mean absorbed doses for the second set of spheres, with volumes ranging from 40 to 3000 mL and an initial activity of 2200 MBq of ^1^ ^7^ ^7^Lu‐DOTATATE, were evaluated using the same methods and showed even stronger statistical agreement. Figure 7a,b present the mean absorbed doses as a function of sphere volume. All three methods demonstrate strong concordance, further highlighting the reliability of the GAN‐generated ^1^ ^7^ ^7^Lu DVKs relative to the Monte Carlo results. Although the GAN DVK slightly overestimated the dose for sphere volumes below 50 mL, it remained largely indistinguishable from both GATE MC and IDAC‐Dose 2.1 for larger volumes.

The GAN‐predicted ^177^Lu DVKs for all the tissues exhibited near‐perfect linear correlation with both GATE Monte Carlo (*r* > 0.99, *p* < 0.001) and IDAC‐Dose 2.1 (*r* > 0.99, *p* < 0.001). These very high correlation coefficients demonstrate that the GAN model successfully captures the dose–volume dependency observed in both reference methods. The paired *t*‐tests presented in Table 2 indicate that, for most tissues, there were no statistically significant differences between the GAN‐predicted and reference mean absorbed doses (*p* > 0.05). However, a small but statistically significant difference was observed for bone in the GATE comparison (*t* = 2.89, *p* = 0.02). Despite this, the overall agreement across all tissues remained strong, as reflected by the high correlation coefficients (*r* > 0.99). This suggests that the observed difference in bone is localized and does not substantially affect the overall method agreement.

**TABLE 2:** Mean absorbed dose correlations between GAN DVK and other methods.

	GATE vs GAN DVK		IDAC vs GAN DVK	
Media	Correlation	*t*‐test	Correlation	*t*‐test
Muscle	*r* = 0.99971	*t* = −1.13, *p* = 0.29	*r* = 0.99944	*t*=−0.70, *p* = 0.50
Water	*r* = 0.99955	*t* = −1.39, *p* = 0.20	*r* = 0.99957	*t* = −1.08, *p* = 0.31
Bone	*r* = 0.99954	*t* = 2.89, *p* = 0.02	*r* = 0.99954	*t* = −1.27, *p* = 0.24
Soft tissue	*r* = 0.99956	*t* = −1.52, *p* = 0.16	*r* = 0.99957	*t* = −1.46, *p* = 0.18

**FIGURE 8:**
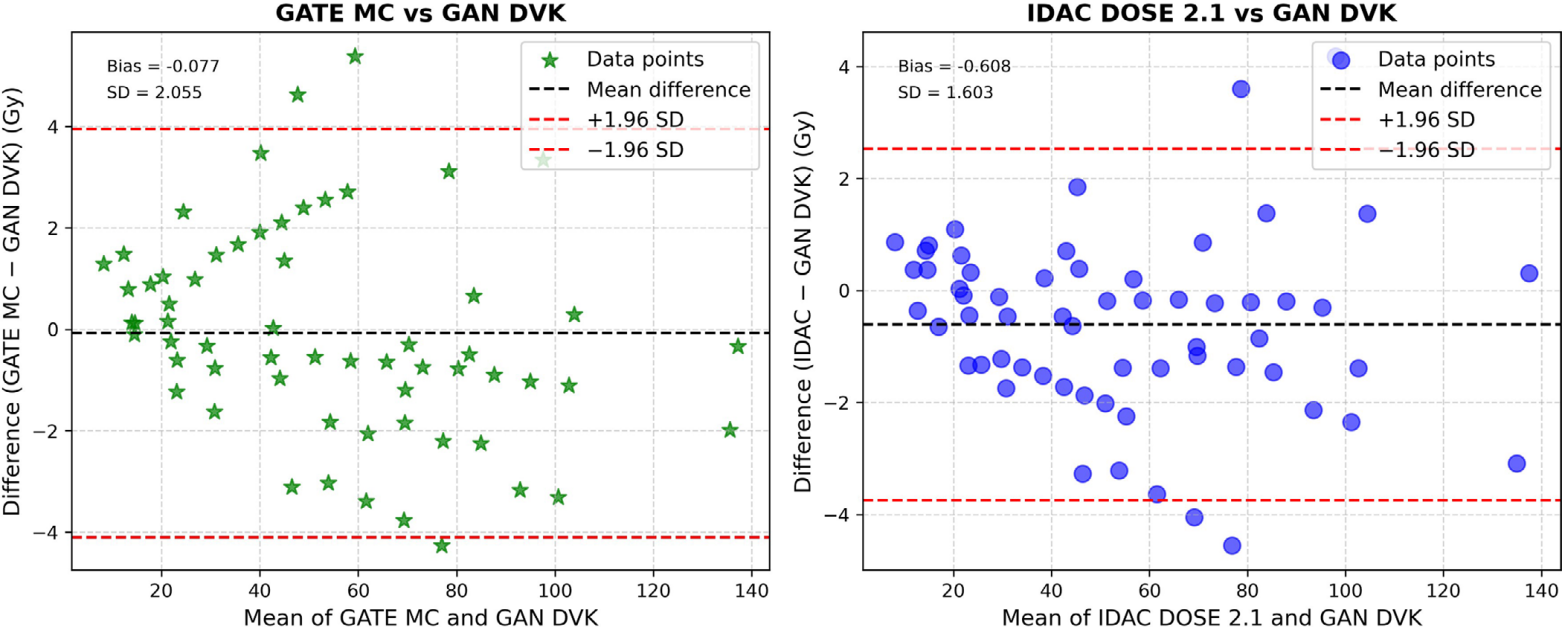
Bland–Altman plots for GATE MC versus GAN DVK (left) and IDAC dose 2.1 versus GAN DVK (right). Over 90% of all points fall within the limits of agreement in both comparisons.

The Bland–Altman analysis comparing GATE MC and GAN DVK in Figure 8 showed a negligible mean bias of −0.077 Gy, with the 95% confidence interval spanning −0.593 Gy–0.438 Gy, indicating no significant systematic difference between the two methods. Although individual measurement differences extended to approximately ± 4 units, as reflected in the limits of agreement (−4.104 Gy to 3.950 Gy) and a standard deviation of 2.055 Gy, this variability did not detract from the overall strong concordance. The confidence intervals around the limits of agreement suggested moderate but acceptable uncertainty, supporting the conclusion that GATE MC and GAN DVK offer highly consistent performance.

In contrast, the comparison between IDAC Dose 2.1 and GAN DVK revealed a modest yet statistically significant mean bias of −0.608 Gy, with a 95% confidence interval of −1.01 Gy to −0.21 Gy, confirming a small systematic difference wherein GAN DVK produces slightly higher estimates. The variability between these two methods was lower, with a standard deviation of 1.60 Gy and limits of agreement ranging from −3.75 Gy to 2.53 Gy, indicating that most differences fell within a narrower band. Overall, the findings demonstrate excellent agreement between GATE MC and GAN DVK, while the comparison with IDAC Dose 2.1 highlights a consistent, quantifiable bias accompanied by tighter dispersion.

**FIGURE 9:**
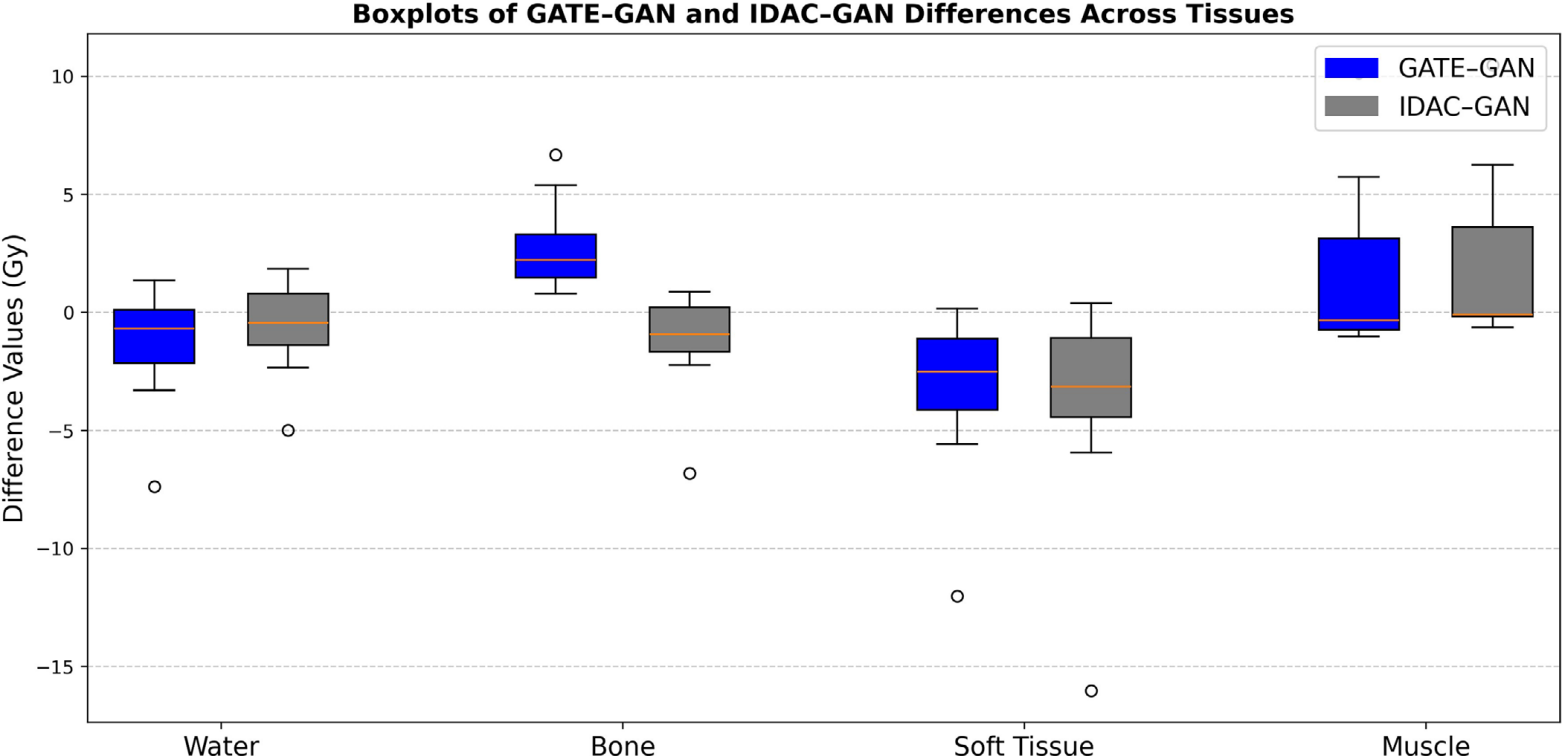
Tissue specific boxplots for comparisons of GAN DVK with GATE MC and IDAC dose 2.1 methods.

The tissue‐level boxplot analyses in Figure 9 reveals additional systematic dose differences between GATE–GAN and IDAC–GAN across distinct tissue types. In water, both approaches demonstrate small mean differences (−1.29 Gy for GATE–GAN and −0.51 Gy for IDAC–GAN) and similar interquartile ranges (IQR ≈ 2.20 Gy), indicating modest variability and comparable performance in low‐density regions. In bone, the methods diverge directionally: GATE–GAN shows a positive mean difference (2.66 Gy), whereas IDAC–GAN exhibits a negative mean difference (−1.08 Gy), though both maintain similar variability (IQR ≈ 1.80–1.90 Gy). These findings underscore that the magnitude and direction of residual discrepancies depend strongly on tissue composition and density.^4^

For soft tissue, both methods show negative mean differences (−2.95 Gy for GATE–GAN and −3.36 Gy for IDAC–GAN) with relatively large IQRs (≈ 3.0–3.3 Gy). Although variability is higher in these mid‐density regions, the close agreement in both central tendency and spread underscores the consistent performance of the GAN‐derived DVKs relative to both MC and IDAC estimates. In muscle, both methods demonstrate positive mean differences (1.22 Gy and 1.71 Gy) along with the largest IQRs across all tissues (≈ 3.8 Gy), reflecting the expected increase in variability within higher‐density soft tissue. Even in this most variable region, the alignment of the central values between GATE–GAN and IDAC–GAN further highlights the stability and reliability of the GAN‐generated DVKs when benchmarked against both reference methods.

## 4 DISCUSSIONS

This study demonstrated an excellent correlation of mean dose calculations (*r* > 0.99) between the GAN‐generated ^177^Lu DVKs and both GATE Monte Carlo (MC) and IDAC Dose 2.1 as references. GAN DVK is therefore a good improvement to the use of single patient‐specific DVK or voxel S‐value applied uniformly across all tissues, which are prone to substantial dosimetric errors. As reported by Cremonesi et al., organ doses computed using organ‐level dosimetry, voxel S‐values, and Monte Carlo generally agreed within approximately ± 7% when MC was used as the reference standard, highlighting the inherent limitations of non–organ‐specific DVKs.^28^ The proposed GAN DVK method balances accuracy and computational efficiency by combining voxel‐level detail with fast computation. While MC offers the highest accuracy at high computational cost and organ‐level methods are fast but less precise, the GAN‐based DVK approach provides a practical middle ground, achieving small differences relative to Monte Carlo with substantially reduced computation time. The adoption of organ‐ or tissue‐specific DVKs can substantially improve dosimetric accuracy.

Despite the strong agreement observed, it is important to contextualize the magnitude of these improvements within the broader uncertainty landscape of ^1^ ^7^ ^7^Lu dosimetry. In current clinical practice, the dominant sources of uncertainty arise from SPECT quantification and activity‐to‐dose conversion, typically on the order of 8–30%.^29^, ^30^ In comparison, the differences introduced by the choice of dose voxel kernel for instance, water‐equivalent versus tissue‐specific are generally smaller and therefore unlikely to independently alter clinical decision‐making at present. However, these differences are systematic rather than stochastic, and may become increasingly relevant as advances in quantitative imaging, reconstruction algorithms, and calibration techniques reduce the overall uncertainty budget. In this context, improving the physical consistency of voxel‐level dose modeling remains an important step toward future accurate patient‐specific dosimetry

Recent advances in DL have further enhanced voxel‐based internal dosimetry. DNNs trained on CT‐derived density maps with MC‐simulated kernels as ground truth have been shown to generate patient‐specific voxel S‐values with high accuracy.^15^ In one such study, a deep neural network achieved a mean relative absolute error (MRAE) of 4.5% ± 1.8% when compared with MC‐derived S‐values.^14^ Similarly, Lee et al. demonstrated that a convolutional neural network (CNN) trained to predict voxel dose‐rate maps achieved voxel‐level errors of 2.54% ± 2.09%, outperforming voxel S‐value convolution (VSV), which produced errors of 9.97% ± 1.79% versus MC.^10^ This poor performance of VSV was largely attributable to the use of non–organ‐specific S‐values, reinforcing the importance of anatomical specificity in voxel dosimetry.

Organ‐averaged methods MIRD/OLINDA‐style^31^ and ICRP‐based tools like IDAC Dose 2.1^24^ were designed to be robust, fast and standardized: they use reference phantoms and precomputed S‐values or specific absorbed fractions (SAFs) to estimate mean absorbed dose to source/target organs from time‐integrated activities. Their advantages are reproducibility, low computational cost, and regulatory acceptance.^24^, ^31^, ^32^, ^33^ However, by design they treat each organ as a spatially homogeneous source and target and therefore cannot capture intraregional heterogeneity in uptake or dose deposition.

Consequences of the homogenization assumption include systematic bias when activity is nonuniform. For instance, focal lesions, cortical versus medullary kidney uptake and loss of clinically important spatial information on hot or cold subregions that may determine efficacy or toxicity.^34^, ^35^ These limitations have been repeatedly noted in comparisons between OLINDA/EXM and voxel methods. IDAC Dose 2.1 and ICRP voxel‐phantom‐based implementations improve on older stylized phantoms by using more anatomically realistic reference voxel phantoms and updated SAFs, but they remain organ‐averaged tools unless combined with patient‐specific activity inputs and additional voxelization workflows.^24^

The relevance of tissue‐specific DVKs is particularly pronounced in anatomically heterogeneous regions and in clinical scenarios involving non‐soft‐tissue lesions. For example, metastatic disease in prostate cancer frequently involves the vertebrae and other osseous structures, where the density and elemental composition differ substantially from water‐equivalent media. In such cases, the use of water‐based kernels may introduce systematic bias in absorbed dose estimation.^33^, ^36^ Tissue‐specific DVKs therefore provide a more physically consistent framework for modeling energy deposition, particularly at interfaces between materials of differing densities, where deviations from homogeneous assumptions are more pronounced.

Voxel dosimetry via Monte Carlo particle transport or precomputed voxel S‐values can explicitly account for patient anatomy and nonuniform activity distributions measured with SPECT/CT or PET/CT.^34^, ^37^, ^38^ It can yield spatial dose maps and metrics like dose‐volume histograms, that are more relevant for heterogeneous dose response problems. However, Monte Carlo calculation faces major computational cost and workflow problems. Comparative studies demonstrate that voxel DVK methods and voxel Monte Carlo are often in good agreement for many radionuclides and voxel sizes but can diverge in small volumes, at boundaries, and for short‐range emitters.^37^, ^39^, ^40^

Finocchiaro et al., compared OLINDA versus voxel kernel convolution versus Monte Carlo. Less mean biases were achieved between voxel convolution versus MC while errors up to 30%–40% were realized in organs. The errors were higher for irregular lesions.^35^ In the present study, while both the Pearson's correlations between IDAC Dose 2.1 vs GAN DVK and GATE vs GAN DVK remain higher (*r* > 0.99), the Bland–Altman bias at 95% confidence interval (CI) are [−1.01 Gy, −0.21 Gy] and [−0.60 Gy, 0.44 Gy] respectively. This reflects an insignificant variability between GAN DVK and GATE Monte Carlo and a slight systemic GAN DVK overestimation against IDAC Dose 2.1. Santoro et al., reported a mean bias of 10%–25% between voxel based PLANET Dose application versus OLINDA/EXM.^41^ The good dosimetric agreement reported by Kim et al., for voxel S‐value (VSV) versus GATE Monte Carlo with mean bias of < 6% suggests that voxel level dosimetry are potentially more accurate than organ level dosimetry.^33^

While the most accurate Monte Carlo methods still place higher computational cost, it is however, important to consider the evolving role of high‐performance computing in internal dosimetry. Recent advances in GPU‐accelerated Monte Carlo simulations are significantly reducing computation times,^42^ and it is plausible that fully patient‐specific Monte Carlo dosimetry may become more clinically accessible in the future. In this context, the proposed GAN‐based approach should be viewed as complementary rather than competitive. Once trained, the model enables near real‐time DVK generation with minimal computational resources, offering advantages in scalability, rapid evaluation, and potential integration into clinical workflows where full Monte Carlo simulations may still be impractical.

Specifically, in the present study, convolution of GAN‐predicted DVKs with a SPECT‐derived TIA map enabled dose map computation in under one minute on a standard CPU‐based workstation (single‐threaded 3.65 GHz dual‐processor system with 16 GB RAM), whereas a Monte Carlo simulation with ∼9  ×  10^7^ primaries typically requires nearly 30 hours on the same workstation. This dramatic speed‐up highlights the practical utility of GAN‐based DVKs for rapid, large‐scale, or workflow‐integrated applications, even as GPU‐accelerated Monte Carlo methods continue to mature.

The present study however, has a few limitations. For instance, the dose calculations were based on regular spherical phantoms with uniform activity distributions unlike in real patient where tissues uptakes can vary. Secondly, the biological elimination was assumed as it is not relevant in phantoms, unlike in real patients where both physical and biological decays play important role to get the correct pharmacokinetics of individual patients. And thirdly, we acknowledge that only a limited set of tissues; water, soft tissue, skeletal muscle, and cortical bone was included in this study. However, these tissues capture the majority of clinically relevant organs and metastatic sites in ^1^ ^7^ ^7^Lu radionuclide therapy. For example, the mass densities of the liver and kidneys closely resemble that of soft tissue, and most neuroendocrine tumor metastases occur in the liver and bones. Therefore, the selected tissues provide a reasonable approximation for initial DVK development and validation, allowing us to assess the feasibility and accuracy of GAN‐predicted tissue‐specific DVKs before extending to additional organ‐specific models in future work.

## 5 CONCLUSIONS

In conclusion, this analysis demonstrates that the GAN DVK method is a viable approach for tissue specific internal radiation dosimetry, showing strong overall agreement with established Monte Carlo and other organ‐style clinical methods. The identified statistical biases between GAN DVK and MC and IDAC Dose 2.1 methods are minimal in the context of the overall distribution and method variabilities. These findings support further development and refinement of DL models like GAN DVK as promising methodological tools for accelerating computationally intensive dosimetry in phantom and future patient‐specific studies. Future work will focus on optimizing the model architecture and training regimen to reduce bias and variability, particularly for outlier cases.

## AUTHOR CONTRIBUTIONS

**Otieno Kapis**: 3D GAN architecture design in python; Manuscript development; Data analysis. **Ian M. Kaniu**: Manuscript coordination; Simulation data analyses. **Iaccarino Giuseppe**: Design of Spherical phantoms; Manuscript coordination. **Hudson Kalambuka Angeyo**: Manuscript topic development; Manuscript coordination.

## CONFLICT OF INTEREST STATEMENT

The authors declare no conflicts of interest.

## Data Availability

All the important end points of the analyzed data are included in this article. The raw data are available from the corresponding author upon reasonable request.
